# Glutamine sustains energy metabolism and alleviates liver injury in burn sepsis by promoting the assembly of mitochondrial HSP60-HSP10 complex via SIRT4 dependent protein deacetylation

**DOI:** 10.1080/13510002.2024.2312320

**Published:** 2024-02-08

**Authors:** Yongjun Yang, Qian Chen, Shijun Fan, Yongling Lu, Qianyin Huang, Xin Liu, Xi Peng

**Affiliations:** a Clinical Medical Research Center, Southwest Hospital, Third Military Medical University (Army Medical University), Chongqing, People’s Republic of China; b State Key Laboratory of Trauma, Burns and Combined Injury, Southwest Hospital, Third Military Medical University (Army Medical University), Chongqing People’s Republic of China

**Keywords:** Glutamine, burn sepsis, HSP60-HSP10 assembly, energy metabolism, mitochondrial electron transport chain, reactive oxygen species, Sirtuin 4, liver injury

## Abstract

Burns and burn sepsis, characterized by persistent and profound hypercatabolism, cause energy metabolism dysfunction that worsens organ injury and systemic disorders. Glutamine (Gln) is a key nutrient that remarkably replenishes energy metabolism in burn and sepsis patients, but its exact roles beyond substrate supply is unclear. In this study, we demonstrated that Gln alleviated liver injury by sustaining energy supply and restoring redox balance. Meanwhile, Gln also rescued the dysfunctional mitochondrial electron transport chain (ETC) complexes, improved ATP production, reduced oxidative stress, and protected hepatocytes from burn sepsis injury. Mechanistically, we revealed that Gln could activate SIRT4 by upregulating its protein synthesis and increasing the level of Nicotinamide adenine dinucleotide (NAD^+^), a co-enzyme that sustains the activity of SIRT4. This, in turn, reduced the acetylation of shock protein (HSP) 60 to facilitate the assembly of the HSP60-HSP10 complex, which maintains the activity of ETC complex II and III and thus sustain ATP generation and reduce reactive oxygen species release. Overall, our study uncovers a previously unknown pharmacological mechanism involving the regulation of HSP60-HSP10 assembly by which Gln recovers mitochondrial complex activity, sustains cellular energy metabolism and exerts a hepato-protective role in burn sepsis.

## 1. Introduction

Burn injuries rank as the fourth most common type of trauma worldwide, yet their profound and enduring effects are frequently overlooked [1, 2]. Individuals with severe burns face heightened vulnerability to infections and sepsis, which are primary culprits behind organ dysfunction and the life-threatening systemic collapse that often culminates in delayed mortality [3]. Patients who experience severe sepsis often exhibit significant liver injury [4, 5], characterized by mitochondrial swelling, structural damage, and functional abnormalities in hepatocytes [6, 7]. Additionally, burn sepsis patients frequently endure prolonged hypercatabolic states [8] and are susceptible to nutritional intolerance. This, in turn, triggers energy metabolism disorders, decreased ATP supply, and increased ROS generation, all of which are crucial factors contributing to the worsening of liver injury [9–12]. Therefore, it may offer novel approaches to tackle this issue by addressing energy metabolism disorders and investigating pharmacological mechanisms that promote a balance between energy demand and nutrient supply.

The preservation of the structural and functional integrity of the electron transport chain (ETC) complexes is of utmost importance. It ensures the smooth flow of electrons, prevents any leakage, and facilitates ATP synthesis without any hindrances [12, 13]. Nutrients act as substrates that drive mitochondrial respiration and maintain the membrane potential. At the same time, the efficiency of protein folding, a process primarily regulated by molecular chaperones within the mitochondria, is crucial for the activity of mitochondrial complexes [14–17]. Among these chaperones, Heat Shock Protein 60 (HSP60) stands out as a highly conserved molecular chaperone that plays a critical role in the import of proteins into the mitochondria and the assembly of macromolecules [18]. In the context of inflammation and sepsis, HSP members has been found to prevent protein aggregation and refold denatured proteins, thereby exhibiting immunomodulatory capabilities [19, 20]. Of particular importance, previous studies have provided evidence that HSP60 plays a crucial role in facilitating the proper folding and assembly of native proteins within the mitochondrial matrix, including the ETC complexes. This is achieved through the formation of chaperonin complexes with HSP10 [18, 21–26]. Furthermore, in critical situations induced by ischemia/reperfusion injury, such as severe burns, the function of the mitochondrial HSP60-HSP10 complex may be compromised, potentially leading to dysfunction within the ETC [17, 27, 28]. Consequently, there may be a potential connection between the HSP complex and energy metabolism, which could serve as an emerging target for pharmacological interventions in the management of sepsis resulting from burn injuries.

Over the past decades, numerous clinical and basic research studies have highlighted the role of glutamine (Gln) in nutritional interventions following burn injury and burn sepsis. These interventions have been shown to effectively reverse malnutrition and improve prognosis [29–31]. However, recent studies have revealed that Gln also possesses non-substrate effects, contributing to the protective mechanisms against burn sepsis through its influence on protein modifications. For instance, Gln regulates cellular metabolism and enhances cellular function through post-translational modifications such as acetylation, succinylation, and O-glycosylation [32, 33]. Notably, Gln acts as a crucial source of Nicotinamide adenine dinucleotide (NAD^+^) and effectively improves the energy metabolism of burn and sepsis patients [8, 34–37]. The SIRT family, a highly conserved NAD ^+ ^-dependent protein deacetylase, plays a significant role in the regulation of NAD ^+ ^. Proteomic studies have provided evidence of the interaction between Sirtuin 4 (SIRT4), located in the mitochondria, and HSP60 [31–33]. In light of this, our study aims to investigate the potential regulatory role of Gln in the activity of HSP60 through SIRT4. Through this exploration, we hope to gain insights into how Gln can help maintain hepatic energy metabolism and alleviate liver injury in burn sepsis. Ultimately, this research will offer a fresh perspective on understanding the pharmacological mechanisms of Gln and its potential benefits in this specific context.

## 2. Materials and methods

### 2.1. Experimental animals

The wild-type BALB/c mice (8-10 weeks) were purchased from the Experimental Animal Center of the Third Military Medical University. The animals were housed in individually ventilated cages under specific pathogen-free conditions and provided with ad libitum food and water one week before the experiment. The animal room was maintained at 25°C with a relative humidity of 40% and a 12-hour light–dark cycle. BALB/c mice were fasted for 12 h before the burn. All animal experiments were conducted in accordance with the National Guidelines for Animal Welfare and approved by the Institutional Animal Ethics Committee of the Third Military Medical University.

### 2.2. Burn models and grouping

According to the previous studies of our research group, a burn sepsis mouse model was established as follows [32, 33, 38–41], mice were randomly divided into three groups: control group, burn sepsis group, and glutamine-treated group (Gln group) with 16 mice in each group. The groups were further divided into two time points, with 8 mice at each time point. After anesthesia, the mice's backs were shaved and placed in a scalding mold, and exposed to 90°C water for 10 s to create a full-thickness skin burn (III°, approximately 15% of the total body surface area). The burn sepsis group and Gln group were immediately subcutaneously injected with Pseudomonas aeruginosa (5.25 × 10^5^ CFU/kg, 27853, American Type Culture Collection, Manassas, VA) at the burn site to establish the burn sepsis model. The back skin of the sham burn group mice was immersed in 37°C water for 10 s to establish the control group. The mice were resuscitated with 50 ml/kg of saline and kept warm in a constant temperature box at 30°C for 24 h. Subsequently, the burned mice were placed in a sterile cage and provided with sterile water and food. The Gln group was injected with 0.25 g/kg glutamine by intraperitoneal injection on the first day, followed by continuous injection of 0.75 g/kg every 24 h for 5 days. The control group was injected with an equal volume of saline. On days 3 and 5 after burn infection, the mice were euthanized by cervical dislocation to obtain peripheral blood and liver tissue.

### 2.3. Cell culture and treatment

The alpha mouse liver 12 (AML-12) was purchased from ATCC (USA). AML-12 cells were cultured in a medium containing 1% ITS supplement (Sigma, USA), 10% fetal bovine serum (Hyclone, USA), and 40 ng/ml dexamethasone (Sigma, USA), and incubated at 37°C in a humidified incubator with 5% CO_2_. AML-12 cells were cultured without (M group) or with 4 mM Gln (Gln group) as controls. For cell treatment, AML-12 were further treated with Lipopolysaccharide (LPS 200 ng/mL, E. coli O55:B5, Sigma, USA) after precondition of different concentrations of glutamine (2, 4, 8 mM, Sigma, USA) for 24 h or not, before the following assays were performed. The M group referred to AML-12 cells cultured without Gln, while the Gln group represented AML-12 cells treated with culture medium containing 4 mM Gln for 24 h. In some experiments, we also added pan- Acetyltransferaseinhibitors (Solarbio, 1-Aminobenzotriazole (1 mM), Curcumin (25μM), EML425 (2μM), China), Nonactin (15 μM, MCE, USA), (E)-Daporinad (NAD^+^ inhibitor, FK866, 0.2 nM, MCE, USA), AZD2461 (NAD^+^ activator, 200 nM, MCE, USA), and β-Nicotinamide mononucleotide (NAD^+^ precursor, NMN, 0.5 mM, MCE, USA) to treat cells together. The selection of NAD^+^ inhibitors, NAD^+^ activators, and NAD^+^ precursor reagents was based on previously published literature [42].

### 2.4. Construction of SIRT4 knockdown AML-12 cells

Mouse SIRT4 knockdown AML-12 cells were constructed using a mouse knockdown virus (Gene, Shanghai, China). Briefly, AML-12 cells (ATCC) were seeded in a 6-well plate at a density of 5× 10^4^ cells/ml, and the virus was added to the plate (multiplicity of infection (MOI)  = 20) for transfection for 24 h. The cells were then cultured in AML-12-specific medium (containing 1% ITS supplement (Sigma), 10% fetal bovine serum (Hyclone, USA), and 40 ng/ml dexamethasone (Sigma, USA)) for 24 h. Subsequently, positive cells were selected by treating them with 2 μg/ml puromycin for 24 h to obtain SIRT4 knockdown AML-12 cells. The knockdown efficiency was determined as 91% by densitometric analysis using western blotting.

### 2.5. Mitochondrial isolation and mitochondrial protein sample preparation

Mitochondria were isolated using kits (sigma, USA, MITOISO2). In brief, 5 × 10^5^ cells were cultured in a 75cm^2^ cell culture flask until reaching a density of about 80-90%. The cells were then digested and enriched, and 200 µL of lysis buffer was added for 5 min to fully lyse the cells. Next, 1X mitochondrial extraction buffer was added and the suspension was centrifuged at 600×g for 10 min at 4°C to separate the cell nuclei and cell debris. The supernatant was collected and centrifuged at 10,000×g for 10 min at 4°C to obtain the mitochondria, which were then added to 100 µL of 1X mitochondrial storage buffer. After using T-PER protein extraction reagent (Thermo Fisher Scientific) containing protease and phosphatase inhibitors to lyse the mitochondria, the protein concentration was determined using the Pierce™ Coomassie Plus Bradford protein assay, followed by western blot analysis. The extraction efficiency was confirmed using protein blotting.

### 2.6. Determination of mitochondrial complex activity

The activities of mitochondrial respiratory chain complex I/NADH-coenzyme Q reductase, mitochondrial complex II/succinate-coenzyme Q reductase, mitochondrial complex III/coenzyme Q-cytochrome c reductase, mitochondrial complex IV/cytochrome c oxidase, and mitochondrial complex V/ATP synthase were measured using commercial test kits (Solarbio, China), with each step carried out according to the manufacturer's instructions. Normalize the data with the control group.

### 2.7. Biological measurement

Biological assays were performed using commercial biochemical assay kits (Nanjing Jiancheng Bioengineering Institute, China). According to the manufacturer's instructions, the enzymatic activities of Alanine transaminase (ALT), Aspartate transaminase (AST), Malondialdehyde (MDA), Myeloperoxidase (MPO), Superoxide dismutase (SOD), and Catalase (CAT) were quantified in serum samples. Enzyme activity normalization was related to the protein content.

### 2.8. HSP60 and HSP10 complex folding assays

The HSP60 and HSP10 folding assays were conducted according to a previous study [43]. The folding tests were performed using the HSP60/HSP10 Glow-FoldTM protein refolding assay kit (Boston Biochem, Cambridge, MA, USA) according to the manufacturer's instructions. In brief, the reaction buffer, HSP60 solution, HSP10 solution, Mg^2+^-ATP solution, Glow-Fold™ Substrate Protein, 10 μM SIRT4 recombinant protein (Cloud-Clone Corp, China), and 50 μM NAD^+^ (Sigma, USA) were combined according to the experimental requirements, with a positive control group set up. The folding reaction was carried out at 30°C for 1, 30, and 60 min, and the relative fluorescence intensity, which reflected folding activity, was measured using a multi-mode microplate reader (Thermo Varioskan Flash, USA) after the addition of the luciferin reagent.

### 2.9. Analysis of mitochondrial protein misfolding

The mitochondrial protein complex misfolding analysis was conducted using a literature-based method [18, 44]. After isolating the mitochondria, NP-40 (1% or 0.1%) lysis buffer was added to each sample. The samples were then incubated on ice for 10 min. The NP-40 insoluble aggregates were precipitated by centrifugation (20000× g) at 4°C and resuspended in NuPAGE LDS sample buffer (Invitrogen, UK). Throughout the protein extraction process, mild lysis conditions were used (no reducing agents, on ice). Subsequently, the proteins were separated on a 4-15% gradient stain-free gel (MP, TGX, Stain-Free, Bio-Rad, USA) and visualized directly using a ChemiDoc XRS^+^ imaging system (Bio-Rad, USA).

### 2.11. Construction of a real-time NAD^+^ probe in cells

A real-time NAD^+^ display probe was constructed through lentivirus based on the method described in reference [38]. After constructing a lentivirus (Gene, Shanghai, China), the virus was added to a 6-well plate (multiplicity of infection (MOI) = 25) and transfected for 24 h. After replacing with AML-12 specific culture medium (containing 1% ITS liquid culture supplement (Sigma, USA), 10% fetal bovine serum (Hyclone, USA), and 40 ng/ml dexamethasone (Sigma, USA)), the cells were cultured for another 24 h. By adding NAD^+^ inhibitors, activators, and precursors, the efficiency of constructing the real-time NAD^+^ probe was determined by observing changes in fluorescence intensity.

### 2.12. Co-immunoprecipitation and western blotting

The processed cells were lysed using T-PER protein extraction reagent (Thermo Fisher Scientific, USA) containing protease and phosphatase inhibitors, and the proteins were separated by SDS-PAGE and transferred onto PVDF membranes. For protein blotting, the antibodies against SIRT4, HSP60, Tubulin, Cytochrome c oxidase IV (COX4, CST, USA), and Pan-acetyllysine antibody (PTM BIO, China) were incubated overnight at 4°C at a dilution of 1:1000. Then, the membranes were incubated with HRP-conjugated secondary antibodies (1:2000) at 37°C for 1 h. The chemiluminescence images were visualized using the Super ECL plus western blotting Substrate kit (Bioground biotechnology, China) and detected using the ChemiDoc XRS^+^ imaging system (Bio-Rad, USA) or Vilber Fusion FX-EDGE imaging system (Grand Paris, France).

For the Co-immunoprecipitation assay, refer to the protocol provided by the supplier for the Co-immunoprecipitation (Co-IP) assay (Thermo Fisher Scientific, USA). In brief, Dynabeads Protein A/G (5 μg) were mixed with SIRT4 or HSP60 antibody (10 μg, CST, USA) and incubated at room temperature for 30 min. Then, 300 μg of mitochondrial protein was added and the mixture was incubated overnight at 4°C with rotation. After thorough washing, the proteins bound to Dynabeads Protein A/G were eluted and denatured with sample buffer, separated by SDS-PAGE, and transferred onto PVDF membranes. The PVDF membranes were blocked with 5% BSA for 1 h and incubated overnight at 4°C with primary antibodies against SIRT4, HSP60, COX4 (CST, USA), HSP10 (abcam, USA) and Pan-acetyllysine antibody (PTM BIO, China) at a dilution of 1:1000. The next day, after washing the membranes with TBST, they were incubated with HRP-conjugated secondary antibodies (1:1000) at room temperature for 1 h. The chemiluminescent signals were detected using the Super ECL plus western blotting Substrate kit (Bioground biotechnology, Chongqing, China) and visualized with the ChemiDoc XRS + imaging system (Bio-Rad) or the Vilber Fusion FX-EDGE imaging system (Grand Paris, France).

### 2.13. The interaction was determined by ITC

Recombinant protein samples of SIRT4 or HSP60 were dissolved in deionized water and vortexed for 15 min, followed by ultrasonic degassing and set aside. The interaction analysis was performed using the Auto ITC200 (GE, USA) microcalorimetry method. 10 μM HSP60 was placed in the titration cell, and 100 μM SIRT4 (Cloud-Clone Corp, China) was placed in the sample cell. In all experiments, a total of 20 titrations were performed, with a single titration volume of 2 μL, a sample injection time of 2 s/μL, and an interval of 120 s to determine the affinity of SIRT4 or HSP60. After completion of the experiment, bad data points were removed for analysis. The MicroCal Origin 5.0 software package was used to analyze the data using a multi-point binding model to determine the equilibrium binding constant (Ka), binding stoichiometry (n), and enthalpy change (ΔH).

### 2.14. Immunofluorescence assay

Mouse liver tissue was frozen and sliced using a Leica CM1950 cryostat (Germany). The samples were then fixed with acetone and incubated overnight at 4°C with primary antibodies against SIRT4 and HSP60 (CST, USA), followed by staining with FITC or Cy3-labeled secondary antibodies (Beyotime, China) for 1 h. Fluorescence images were collected using a laser scanning confocal microscope (LSM880; Zeiss, Germany).

### 2.15. Hematoxylin–eosin staining (HE)

The liver tissue obtained from mice was fixed in 4% paraformaldehyde, embedded in paraffin, and cut into 5 μm sections. Subsequently, the paraffin-embedded tissue sections were stained with hematoxylin and eosin and analyzed using a Zeiss optical microscope (AX10 IMAGER.A2, Zeiss, Germany).

### 2.16. Mitochondrial membrane potential detection

Mitochondrial membrane potential was measured using a commercial mitochondrial membrane potential detection kit (TMRE) (Beyotime, China) for hepatocytes, following the manufacturer's instructions. Briefly, after cell treatment, TMRE was incubated with cells at 37°C for 15 min. The supernatant was then removed and cells were washed twice with preheated cell culture medium. After adding 2ml of preheated cell culture medium, fluorescence images were collected using a laser scanning confocal microscope (LSM880; Zeiss, Germany).

### 2.17. ATP assay

ATP content in cells was measured using a commercial enhanced ATP assay kit (Beyotime, China), following the manufacturer's instructions. Briefly, the isolated cell lysates or standard samples were mixed with 100 μL of ATP detection working solution and quickly mixed. The intensity of the luminometer was measured using a multifunctional reader (Thermo Varioskan Flash, USA).

### 2.18. NAD^+^/NADH assay

To determine the ratio of NAD^+^/NADH in cells, a commercially available NAD^+^/NADH quantification kit (Sigma-Aldrich, USA) was used following the manufacturer's instructions. After cell treatment, the cell pellet was suspended in 200 μL of NAD^+^/NADH extraction buffer and subjected to two freeze–thaw cycles on dry ice for 20 min, followed by centrifugation at 13000×g for 10 min. The supernatant was divided into two portions, one for measuring NAD^+^ total, and the other was treated at 60°C for 30 min to fully consume NAD ^+ ^. Subsequently, the absorbance at 450nm was measured. The amount of oxidized NAD^+^ was obtained by subtracting the value of NADH. The ratio of NAD^+^/NADH was calculated using the formula NAD^+^/NADH.

### 2.19. ROS assay

To quantify the level of ROS in cells, a cytoplasmic ROS indicator, DCFH-DA (10 μM, Sigma), was used. After treatment with LPS alone or in combination with different doses of Gln (2 mM, 4 mM, 8 mM) for 24 h, the cells were incubated with DCFH-DA for 15 min. The samples were then washed with pre-cooled washing solution and the level of cytoplasmic ROS was measured using an ACEA NovoCyte flow cytometer (San Diego, CA, USA).

### 2.20. Apoptosis detection

To measure cell apoptosis, the Annexin V-FITC apoptosis detection kit (BD Biosciences, USA) was used. Specifically, cells were seeded at a density of 1 × 10^5^ cells in culture flasks. After treatment with LPS alone or in combination with different doses of Gln (2 mM, 4 mM, 8 mM) for 24 h, the cells were collected by trypsin digestion. The cells were resuspended in 500 μL of 1× binding buffer and incubated with 5 μL of Annexin V-FITC for 5 min. After washing the cells to remove excess probe, 10 μL of propidium iodide (PI) was added and the cells were incubated for 10 min. The cells were then washed again to remove excess probe. Blank control and single staining control groups were set up. The number of apoptotic cells was detected using a flow cytometer (ACEA NovoCyte, USA).

### 2.21. Statistical analysis

The statistical analysis of experimental results was conducted using GraphPad Prism v.8.0.1 software (GraphPad, La Jolla, CA). The normality of data distribution was assessed by performing the Shapiro–Wilk test. The measurement data were expressed as mean ± standard deviation (SD). The two groups were compared using the student’s t-test. For comparisons among multiple groups, one-way ANOVA with post-hoc Bonferroni correction was used. All analyses were considered statistically significant at *P* < 0.05.

## 3. Results

### 3.1. Glutamine alleviates tissue injury, promotes redox balance, and restores ATP generation in the liver of burn septic mice

**Figure 1.**
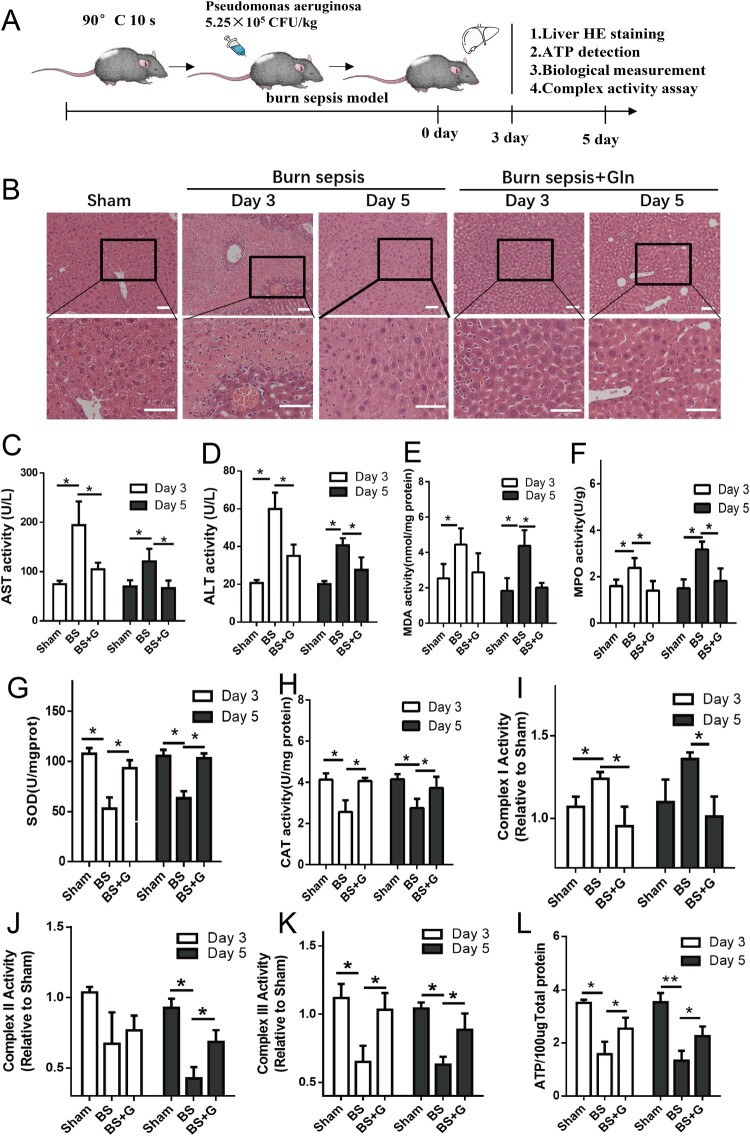
Gln alleviates tissue injury, promotes redox balance, and restores ATP generation in the liver of burn septic mice**. (A)** Time flow of burn sepsis model construction and treatment process. **(B-D)** The pathological changes of mouse liver at 3 and 5 days with or without Gln treatment for burn sepsis **(B)**, and changes in ALT **(C)** and AST **(D)** levels in peripheral blood of burn septic mice with or without Gln treatment. **(E-K)**. Changes in malondialdehyde **(E)**, myeloperoxidase **(F)**, superoxide dismutase **(G)**, catalase **(H)**, mitochondrial complex I **(I)**, mitochondrial complex II **(J)**, and mitochondrial complex III **(K)** activities in liver at 3 and 5 days with or without Gln treatment for burn sepsis. **(L)** Changes in ATP levels in liver at 3 and 5 days with or without Gln treatment for burn sepsis. n ≥ 3 per group (Sham: n = 8, BS3: n = 4; BS5: n = 3; BS3 + G: n = 5; BS5 + G: n = 5), * *P* < 0.05, ** *P* < 0.01, Scale bar: 50 μm. Sham: sham burn group, BS (Burn Sepsis): burn sepsis group, BS + G (Burn Sepsis + Gln): burn sepsis + glutamine group.

To investigate the potential of glutamine (Gln) in ameliorating liver injury resulting from burn sepsis, we established a murine scald infection model and administered Gln via intraperitoneal injection. Figure 1A depicts the process of establishing the burn sepsis model and the detection of markers that reflect liver injury post burn sepsis. Histopathological examination revealed extensive necrosis, accompanied by hepatocyte swelling and enlargement, as well as disruption of the liver lobule structure and disappearance of the radiating structure in the liver tissue, which occurs on the 3rd day after the onset of burn sepsis and persisted until the 5th day (Figure 1B). The serum levels of ALT and AST were also significantly elevated, indicating the severity of liver damage following burn sepsis (Figure 1C-D). Gln supplementation significantly reduced liver necrosis and inhibited the release of AST and ALT in peripheral blood, suggesting its protective roles against liver damage caused by burn sepsis (Figure 1C-D). In addition, burn sepsis disrupted the liver’s redox balance by favoring an oxidative state, as indicated by the upregulation of MDA and MPO and the downregulation of SOD and CAT in liver tissues. Gln significantly restored the redox balance in the liver by inhibiting the increase of MDA and MPO and the decline of SOD and CAT (Figure 1E-H). Given that the redox status is closely related to electron transfer, we measured the activity of mitochondrial complexes I, II, and III in the liver. As expected, the activity of mitochondrial complex I (one of the key sites of electron leakage) was increased while the activity of mitochondrial complexes II and III was decreased. Accordingly, Gln could inhibit complex I while restoring the activity of complexes II and III (Figure 1I-K), leading to an elevated ATP generation (Figure 1L). It is noteworthy that the activity of complex II did not decrease significantly on the third day after modeling. Accordingly, Gln did not remarkably restored it activity. We speculate that this may be due to a relatively lower electron leakage of complex II compared to complex III, leading to a reduced demand of protection by Gln. Overall, these results suggest that the ETC complex was disrupted post burn injury while Gln can maintain the activity of mitochondrial complexes in the liver, promote liver energy metabolism balance, and thereby alleviate liver damage.

### 3.2. Glutamine reduces oxidative stress, maintains cellular redox balance, and promotes ATP production in hepatocytes

**Figure 2.**
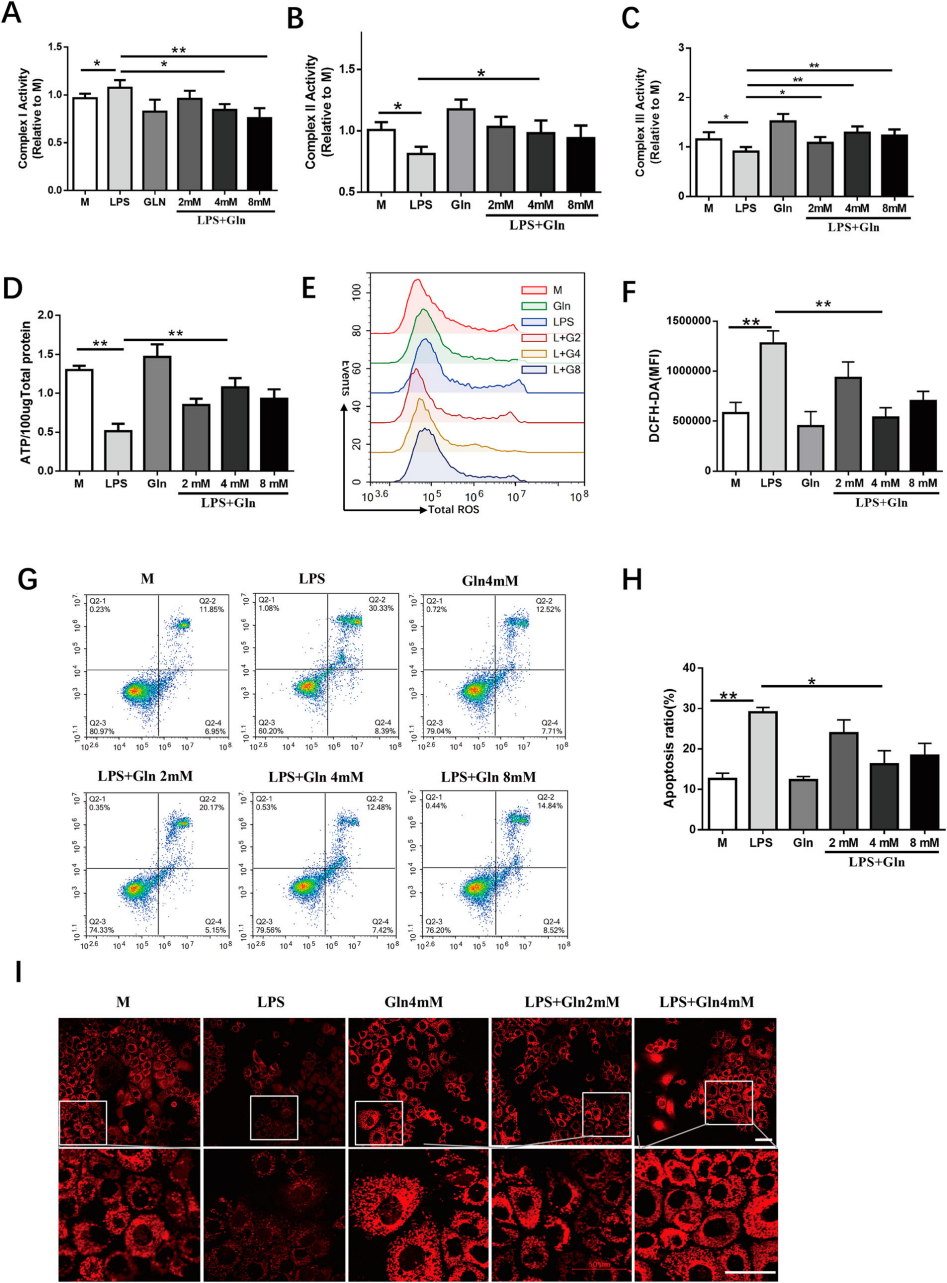
Gln reduces oxidative stress in hepatocytes, maintains cellular homeostasis, and promotes ATP production. **(A-C)** The effects of LPS alone or in combination with varying doses of Gln (2, 4, 8 mM) on the activity of mitochondrial complexes I **(A)**, II **(B)**, and III **(C)** in AML-12 cells. **(D-I)** The changes in ATP **(D)**, total ROS **(E-F)**, cell apoptosis **(G, H)**, and mitochondrial membrane potential **(I)** after treatment with LPS (200 ng/mL) alone or in combination with varying doses of Gln (2, 4, 8 mM) in AML-12 cells. n = 3. Scale bar: 50 μm. * *P* < 0.05, ** *P* < 0.01.

To investigate Gln's direct protective effect on hepatocytes, we established a hepatocyte injury model in vitro by treating AML-12 cells with LPS, which simulates liver damage caused by burn sepsis. Our results showed that LPS increased mitochondrial complex I activity (Figure 2A) while decreasing mitochondrial complexes II and III activity (Figure 2B, C), consistent with alterations observed in liver tissue from the burn sepsis model. Gln inhibited complex I activity but enhanced complexes II and III activity (Figure 2A-C). Furthermore, Gln reversed the decrease in ATP production induced by LPS in hepatocytes (Figure 2D). To clarify whether Gln balanced hepatocyte's oxidative-reductive state to maintain ETC complex integrity, we measured ROS, mitochondrial membrane potential, and cell viability in AML-12 cells. Our results showed that LPS led to a significant increase in ROS (Figure 2E-F), loss of mitochondrial membrane potential (Figure 2I), and cell apoptosis induction (Figure 2G-H). However, Gln reduced total ROS production, maintained mitochondrial membrane potential, and inhibited LPS-induced cell apoptosis (Figure 2E-I). These findings indicate that Gln can maintain mitochondrial energy metabolism's steady state and reduce oxidative stress occurrence in hepatocytes.

### 3.3. Glutamine maintains the activity of mitochondrial complexes for ATP production via HSP60-HSP10 dependent reduction of mitochondria protein misfolding

**Figure 3.**
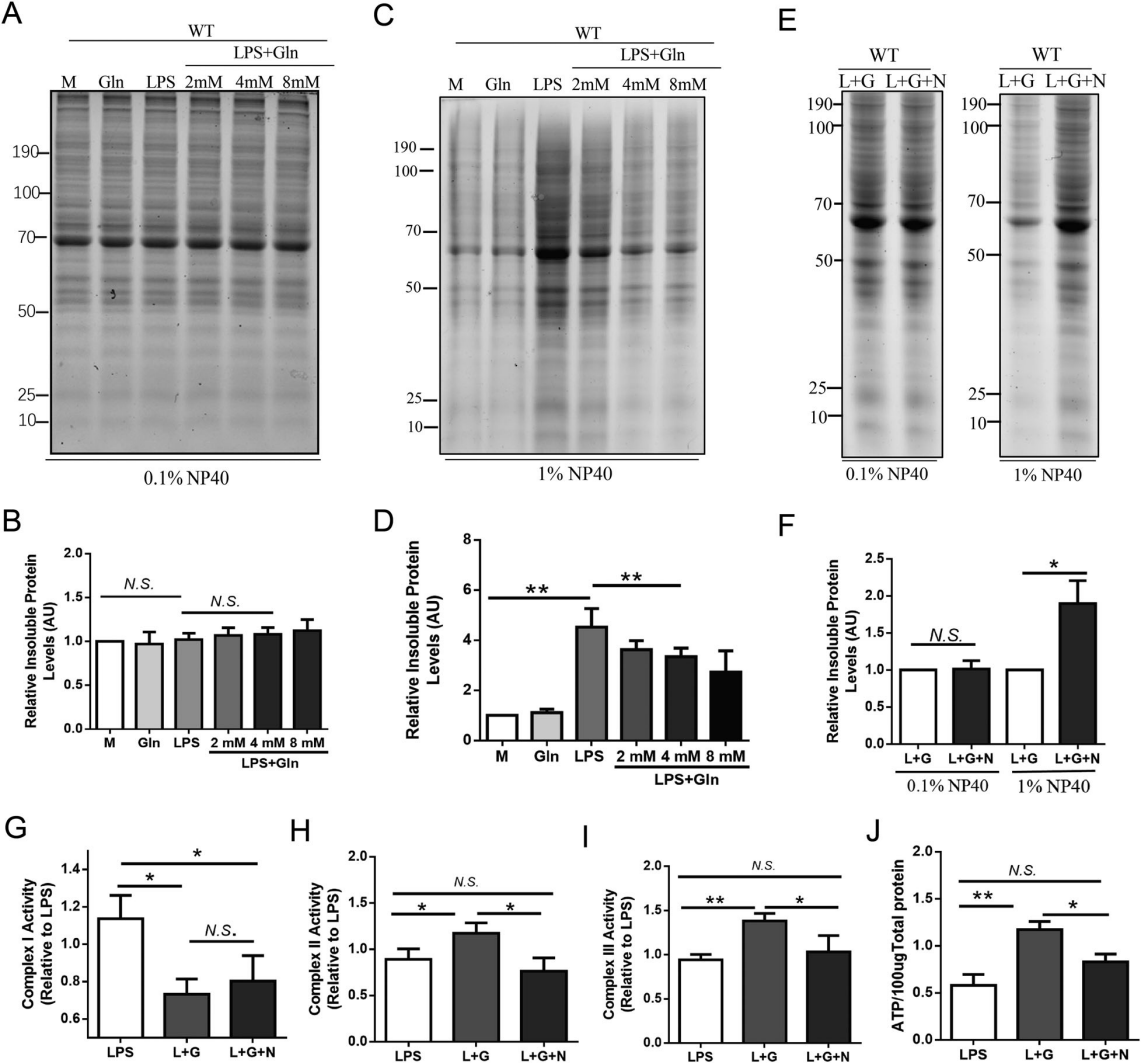
Glutamine maintains the activity of mitochondrial complexes for ATP production via HSP60-HSP10 dependent reduction of mitochondria protein misfolding. **(A-D)** AML-12 cells were treated with LPS (200 ng/mL) alone or in combination with different doses (2, 4, 8 mM) of Gln. Mitochondria were isolated and evaluated for the effect of different treatments on total mitochondrial protein misfolding using SDS-PAGE (4-15% gradient unstained) gels, after treatment with 0.1% **(A, B)** and 1% **(C, D)** Nonidet P-40. **(E-F)** Mitochondrial proteins were extracted from wild-type AML-12 cells treated with LPS (200 ng/mL), Gln (4 mM), and HSP60 inhibitor (15 μM, Nonactin) alone or in combination, and the separation of insoluble aggregates after treatment with 0.1% and 1% **(E, F)** Nonidet P-40 was evaluated using SDS-PAGE (4-15% gradient stain-free) gels to determine the effect of HSP60 inhibition on total mitochondrial protein misfolding. **(G-I)**. Changes in mitochondrial complexes I **(G)**, II **(H)**, and III **(I)** were evaluated in AML-12 cells treated with LPS (200 ng/mL), HSP60 inhibitor (15 μM, Nonactin), or Gln (4 mM) alone or in combination. **(J)** Changes in ATP content were evaluated in AML-12 cells treated with LPS (200 ng/mL), HSP60 inhibitor (15 μM, Nonactin), or Gln (4 mM) alone or in combination. AU indicates arbitrary units with M or LPS + Gln normalized to 1, showing the relative changes in mitochondrial proteins after LPS treatment of AML-12 cells with different doses of Gln. n = 3. * *P* < 0.05, ** *P* < 0.01, *N.S*. no significance.

Correct folding of mitochondrial proteins is crucial for maintaining cellular energy metabolism balance, as demonstrated in multiple studies [16, 18]. To investigate protein misfolding conditions in injured hepatocyte mitochondria, we isolated mitochondria from hepatocytes and prepared total mitochondrial protein (Figure S1B). We analyzed detergent-insoluble mitochondrial proteins to determine if Gln could improve mitochondrial protein misfolding [18]. Mild conditions (0.1% Nonidet P-40) did not significantly increase the insoluble protein of total mitochondrial proteins (Figure 3A, B). However, using a more stringent detergent (1% Nonidet P-40), the misfolded protein in the Gln group was reduced by approximately 40% compared to the LPS group (Figure 3C, D). These results suggest that Gln can reduce mitochondrial protein misfolding. Molecular chaperones, such as HSP60-HSP10, play a crucial role in preserving the correct folding of mitochondrial complexes and maintaining their activity [18, 21–23]. Therefore, we used an HSP60 inhibitor (Nonactin) to interfere with the activity of the HSP60-HSP10 complex (Figure 3E-F) and determine whether Gln maintains the activity of mitochondrial complexes for ATP production by regulating HSP60-HSP10. The results showed that Nonactin abolished Gln's effect to reduce mitochondrial protein misfolding in AML-12 cells. Correspondingly, Nonactin also interrupted the ability of Gln to enhance the activity of mitochondrial complexes II and III (Figure 3G-I) and promote ATP production (Figure 3J). These results suggest that Gln may promote HSP60-HSP10-dependent mitochondrial protein misfolding to maintain the activity of mitochondrial complexes for ATP generation.

### 3.4. Glutamine reduces protein misfolding by repressing the acetylation of HSP60

**Figure 4.**
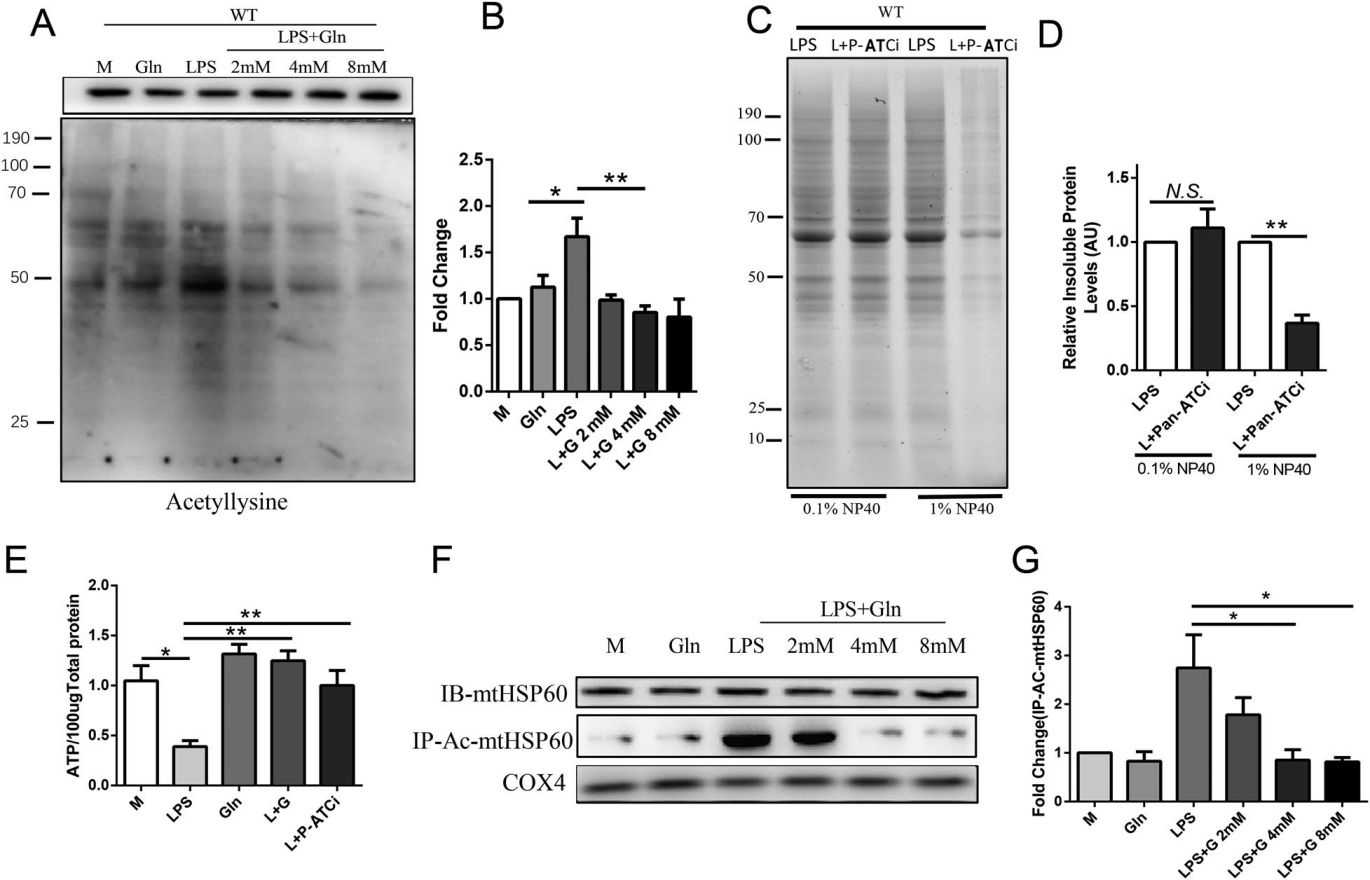
Glutamine reduces protein misfolding by inhibiting the acetylation of HSP60. **(A-B)** AML-12 cells were treated with LPS alone or in combination with different doses (2, 4, 8 mM) of Gln, and mitochondria were extracted and probed with a pan-antibody to detect changes in acetylation modification in the cells. **(C-D)** AML-12 cells were treated with LPS (200 ng/mL) and pan-Acetyltransferase inhibitors (Pan-ATCi) alone or in combination, and mitochondria were isolated and the insoluble aggregates were evaluated using SDS-PAGE (4-15% gradient stain-free) gels after treatment with different concentrations of Nonidet P-40 to determine the effect of different treatments on total mitochondrial protein misfolding. **(E)** Changes in ATP content in AML-12 cells treated with LPS alone, Gln alone, or in combination with pan-Acetyltransferase inhibitors (Pan-ATCi). **(F-G)** AML-12 cells were treated with LPS (200 ng/mL) alone or in combination with different doses of Gln (2, 4, 8 mM), and then the mitochondria were extracted to detect the protein expression of HSP60. After enriching the HSP60 protein using CO-IP, the acetylation modification of HSP60 was detected using an anti-acetylation antibody. AU represents arbitrary units with M or LPS + Gln normalized to 1, showing the relative changes in mitochondrial proteins after treatment of AML-12 cells with LPS in combination with Pan-ATCi. n = 3. * *P* < 0.05, ** *P* < 0.01, *N.S*. no significance.

Recent studies have revealed that mitochondrial activity is impaired by excessive acetylation of mitochondrial proteins [45]. The SIRT family has been identified as a substrate of the HSP60-HSP10 complex, which catalyzes protein deacetylation [18, 46]. In this study, we investigated the changes in total mitochondrial protein acetylation in LPS-treated hepatocytes with or without Gln. Our results showed that LPS enhanced the pan-acetylation of mitochondrial proteins in AML-12 cells, while Gln and pan- Acetyltransferase inhibitors (Pan-ATCi) reduced it (Figure 4A-D). Additionally, Gln and Pan-ATCi stimulated ATP generation, indicating that inhibiting protein acetylation could restore energy metabolism in hepatocytes (Figure 4E). We speculated that HSP60 may be directly acetylated, considering that both HSP60 inhibitors (Nonactin) and Pan-ATCi can affect the assembly of mitochondrial complexes. As expected, Gln did not affect the total protein levels of HSP60 in the mitochondria of AML-12 cells (Figure 4F-G). However, Gln significantly inhibited the level of acetylated HSP60, which was upregulated by LPS treatment (Figure 4F-G). These findings suggest that Gln can alleviate protein misfolding and promote ATP production by inhibiting the acetylation of mitochondrial HSP60.

### 3.5. Glutamine enhances the assembly of the HSP60-HSP10 complex through deacetylation of HSP60 by SIRT4

**Figure 5.**
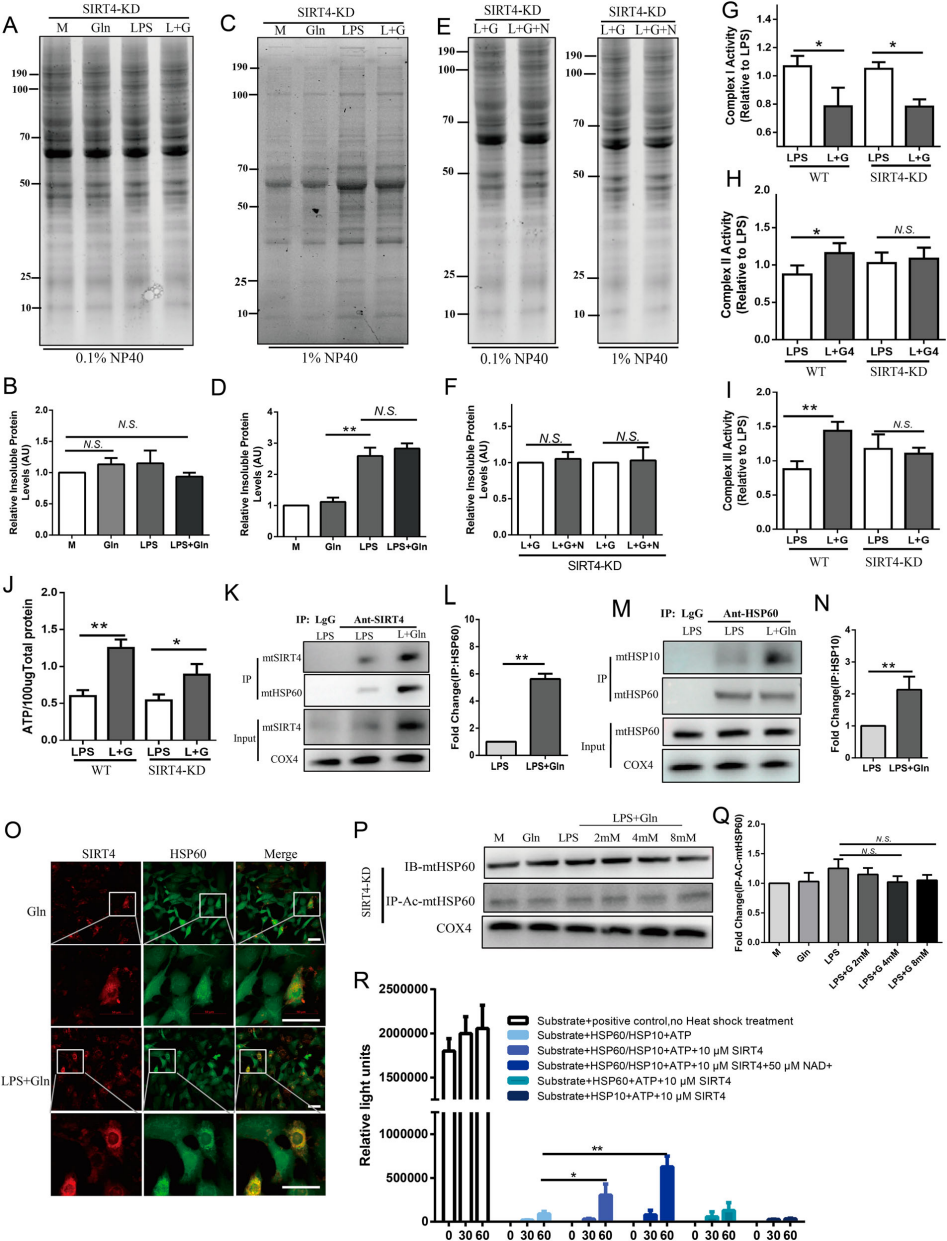
Gln enhances the assembly of the HSP60-HSP10 complex through deacetylation of HSP60 by SIRT4. **(A-D)** Mitochondrial proteins were extracted from SIRT4-KD AML-12 cells treated with LPS (200 ng/mL) and Gln (4 mM) alone or in combination, and the separation of insoluble aggregates after treatment with 0.1% **(A, B)** and 1% **(C, D)** Nonidet P-40 was evaluated using SDS-PAGE (4-15% gradient stain-free) gels to determine the effect of different treatments on total mitochondrial protein misfolding. **(E-F)** Mitochondrial proteins were extracted from SIRT4-KD AML-12 cells treated with LPS (200 ng/mL), Gln (4 mM), and HSP60 inhibitor (15 μM, Nonactin) alone or in combination, and the separation of insoluble aggregates after treatment with 0.1% **(E)** and 1% **(F)** Nonidet P-40 was evaluated using SDS-PAGE (4-15% gradient stain-free) gels to determine the effect of SIRT4-KD and HSP60 inhibition on total mitochondrial protein misfolding. **(G-I)** Changes in mitochondrial complexes I **(G)**, II **(H)**, and III **(I)** were evaluated in WT and SIRT4-KD AML-12 cells treated with LPS (200 ng/mL) or Gln (4 mM) alone or in combination. **(J)** Changes in ATP were evaluated in WT and SIRT4-KD AML-12 cells treated with LPS (200 ng/mL) or Gln (4 mM) alone or in combination. **(K-L)** AML-12 cells were treated with Gln alone or in combination with LPS, and then the interaction between SIRT4 and HSP60 was detected in vivo using CO-IP technology. **M-N**. AML-12 cells were treated with Gln alone or in combination with LPS, and then the interaction between HSP10 and HSP60 was detected in vivo using CO-IP technology. **(O)** AML-12 cells were treated with Gln (4 mM) alone or in combination with LPS (200 ng/mL), and then the co-localization of SIRT4 and HSP60 was observed using immunofluorescence technology. **(P-Q)** AML-12 SIRT4-KD cells were treated with LPS (200 ng/mL) alone or in combination with different doses of Gln (2, 4, 8 mM), and then the mitochondria were extracted to detect the protein expression of HSP60. After enriching the HSP60 protein using CO-IP, the acetylation modification of HSP60 was detected using an anti-acetylation antibody. **(R)** Using assay kits to determine the effects of SIRT4 and NAD^+^ on HSP60-HSP10 assembly. AU indicates arbitrary units with M or LPS + Gln normalized to 1, showing the relative changes in mitochondrial proteins after treatment of SIRT4-KD AML-12 cells with LPS alone or in combination with Gln. n = 3. Scale bar: 50 μm. * *P* < 0.05, ** *P* < 0.01, *N.S*. no significance.

SIRT4, a mitochondrial-specific deacetylase, inhibits protein acetylation. Proteomics research has identified HSP60 as a candidate substrate of SIRT4 [46–48]. To investigate whether SIRT4 contributes to the reduced misfolding of mitochondrial proteins induced by Gln, we knocked down SIRT4 in AML-12 cells and determined its efficiency through Western Blot analysis (Figure S1A). We then analyzed the effect of Gln on mitochondrial protein folding in SIRT4-KD hepatocytes. As expected, knockdown of SIRT4 reversed Gln's ability to reduce mitochondrial protein misfolding (Figure 5A-D). Furthermore, the HSP60 inhibitor (Nonactin) could not abolish the ability of Gln to reduce mitochondrial protein misfolding in SIRT4-KD hepatocytes, suggesting that SIRT4 may act upstream of HSP60 (Figure 5E, F). Although SIRT4 knockdown had a minor effect on the activity of mitochondrial complex I, it reversed the enhanced activity of mitochondrial complexes II and III, which LPS impaired (Figure 5G-I). The knockdown of SIRT4 had an impact on ATP production as well (Figure 5J). The Gln-induced lysine acetylation modification of mitochondrial HSP60 protein was no longer effective in SIRT4-KD AML-12 cells (Figure 5P-Q), indicating that HSP60 may be a target of SIRT4 deacetylase. The ITC assay revealed a direct interaction between HSP60 and SIRT4 (Figure S2), and immunofluorescence experiments demonstrated the co-localization of mtHSP60 and SIRT4 (Figure 5O). CO-IP experiments further confirmed the interaction between mtHSP60 and SIRT4 (Figure 5K-L). Co-immunoprecipitation experiments showed that Gln promoted the interaction between HSP60 and HSP10 (Figure 5M-N), suggesting that Gln facilitates the assembly of the HSP60-HSP10 complex. Furthermore, we directly observed the assembly of the HSP60-HSP10 complex in the presence or absence of SIRT4. We found that the addition of SIRT4 promoted the assembly of the HSP60-HSP10 complex, and this effect was more pronounced in the presence of NAD+, a coenzyme of SIRT4 (Figure 5R). These findings suggest that Gln promotes the deacetylation of HSP60 through a SIRT4-dependent direct interaction mechanism, which in turn enhances the assembly of the HSP60-HSP10 complex required for the proper functioning of the ETC complex, ultimately favoring ATP generation.

### 3.6. Glutamine promotes NAD^+^ generation to activate SIRT4 and sustain the activity of ECT complex for ATP production

**Figure 6.**
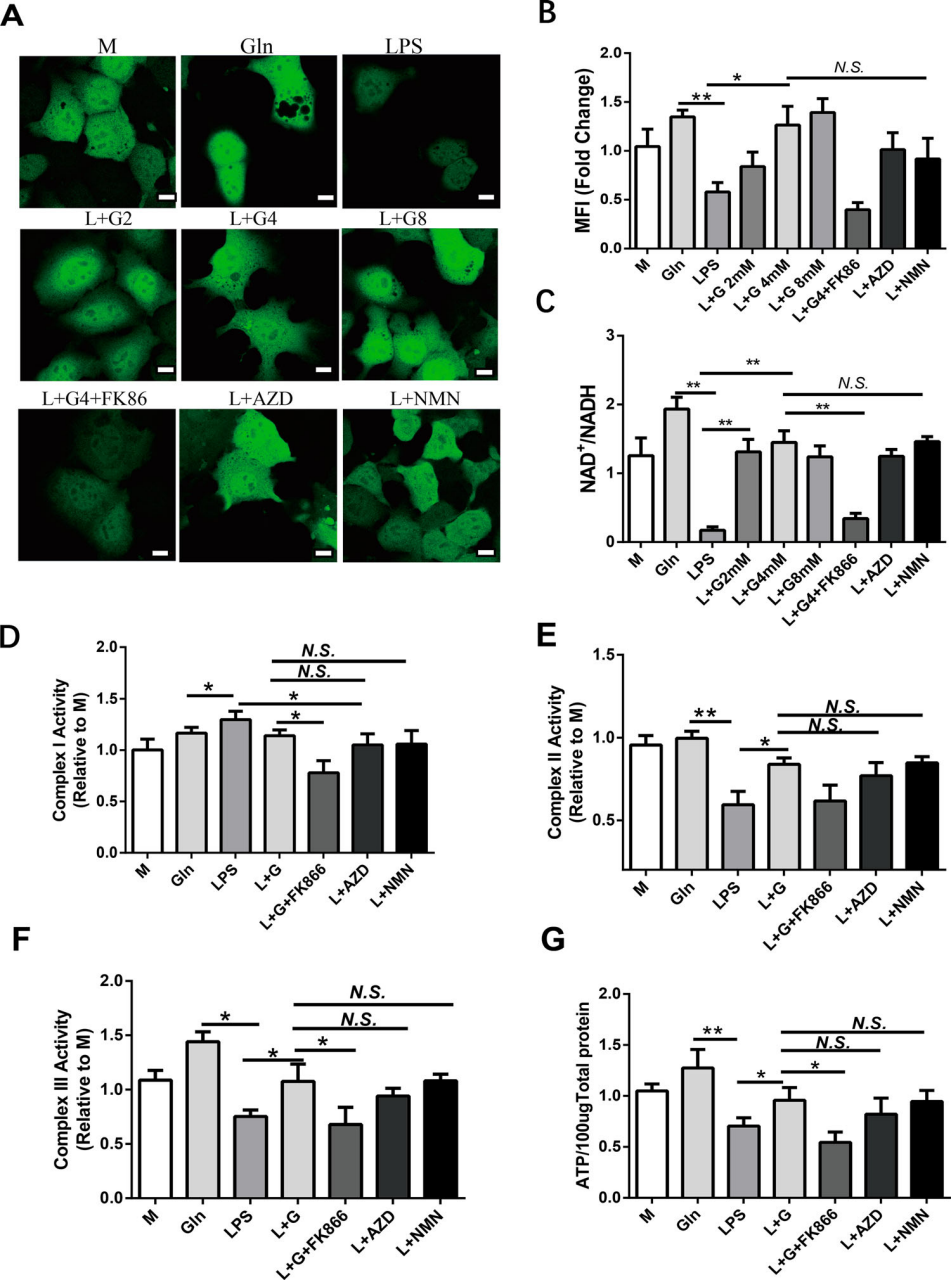
Gln maintains the activity of mitochondrial ETC complexed by inducing NAD^+^ production. **(A-B)** A fluorescent probe was constructed to monitor NAD^+^ in real-time, and the changes in NAD + were determined in AML-12 cells treated with LPS (200 ng/mL) alone or in combination with different doses of Gln (2, 4, 8 mM), as well as in AML-12 cells treated with NAD^+^ inhibitors (FK86, 0.2 nM), activators (AZD2461, 200 nM), and precursors (NMN, 0.5 mM). **(C)** Changes in NAD^+^/NADH were detected in AML-12 cells treated with LPS (200 ng/mL) alone or in combination with different doses of Gln (2, 4, 8 mM), as well as in hepatocytes (AML-12) treated with NAD^+^ inhibitors (FK86), activators (AZD2461), and precursors (NMN). **(D-F)** Changes in mitochondrial complexes I **(D)**, II **(E)**, and III **(F)** were evaluated in AML-12 cells treated with LPS (200 ng/mL) alone or in combination with Gln (4 mM), NAD^+^ inhibitors (FK86), activators (AZD2461), and precursors (NMN). **(G)** The changes in ATP were evaluated in AML-12 cells treated with LPS (200 ng/mL) alone or in combination with Gln (4 mM), NAD^+^ inhibitors (FK86), activators (AZD2461), and precursors (NMN). n = 3. Scale bar: 10 μm.* *P* < 0.05, ** *P* < 0.01, *N.S*. no significance.

SIRT4 relies on NAD^+^ to fully exert its deacetylase activity, and Gln is recognized as a pivotal alternative substrate for NAD^+^ generation in mitochondrial energy metabolism. Therefore, we explored whether Gln activated SIRT4 by inducing NAD^+^ generation. By utilizing an NAD^+^ probe that could monitor real-time changes in NAD+, we observed a reduction of NAD^+^ by LPS treatment, which was reversely upregulated by additional Gln treatment (Figure 6A, B). Furthermore, Gln increased the ratio of NAD^+^/NADH, which was reduced by LPS treatment (Figure 6C, D). To further elucidate the role of NAD^+^ in energy metabolism, we replaced Gln with an NAD^+^ activator (AZD) or a precursor (NMN), or co-treated Gln with an NAD^+^ synthesis inhibitor (FK86), which resulted in either an increase (AZD and NMN) or inhibition (Gln + FK86) of NAD^+^ generation (Figure 6A-D). Accordingly, NAD^+^ activator (AZD) and precursor (NMN) decreased the activity of complex I to reduce ROS generation and significantly maintained the activity of complexes II and III to promote the efficiency of electron transfer (Figure 6D-F). AZD and NMN also promoted the generation of ATP (Figure 6G). In contrast, FK86 markedly abolished the effect of Gln to maintain the activity of ECT complexes and promote ATP production. Collectively, our findings indicate that Gln promotes NAD^+^ generation to activate SIRT4 and sustain the activity of ECT complex for ATP production.

## 4. Discussion

It is widely recognized that glutamine plays a crucial role as a vital nutritional substrate in response to various cellular demands during stress conditions, such as burns and burn sepsis [8, 36, 49, 50]. However, its potential pharmacological effects on sustaining mitochondrial function and preserving energy metabolism remain unclear. Our current study has unveiled that glutamine effectively rescues the dysfunctional mitochondrial electron transport chain (ETC) complex, thereby restoring energy metabolism, reducing oxidative stress, and protecting hepatocytes from burn sepsis injury. Mechanistically, these effects are achieved through the upregulation and activation of SIRT4, which subsequently reduces the acetylation of HSP60 and facilitates the assembly of the HSP60-HSP10 complex. The intact HSP60-HSP10 complex plays a crucial role in sustaining the activity of mitochondrial ETC complex II and III, ultimately promoting the generation of ATP.

The burn sepsis animal model is a classic model, typically established based on indicators such as inflammation and organ damage. The establishment of the burn sepsis model in this study was based on previous research [38–40] and was appropriately modified. In our previous studies, we primarily evaluated the establishment of the burn sepsis animal model through the expression of inflammatory factors, liver function tests, renal function tests, and liver histopathology [41]. Numerous studies, including those conducted in our laboratory [32, 33], have unequivocally demonstrated the pharmacological efficacy of glutamine in treating burns and burn sepsis. Glutamine replenishes the intermediate of α-ketoglutarate required for the tricarboxylic acid cycle, thereby preserving systemic energy metabolism [36, 51]. Furthermore, additional research has shed light on the antioxidant and immunomodulatory properties of glutamine, which help alleviate the detrimental effects of oxidative stress and excessive inflammation [52, 53]. Although the use of glutamine in burn sepsis remains somewhat controversial, clinical nutrition guidelines from various countries recommend its administration to burn patients [54–57]. Analysis has shown that glutamine plays a crucial role in alleviating cellular metabolic disorders, reducing the high metabolism following burns, mitigating organ damage, and promoting tissue repair [58–60]. Therefore, we believe that while glutamine may not shorten the discharge time for surviving patients, it can reduce the damage to individual tissue organs caused by cellular metabolic disorders, such as the intestine and liver. In our current study, we have advanced our understanding of how glutamine mitigates oxidative stress and excessive inflammatory response via a direct role to sustain energy metabolism. Specifically, we have discovered that glutamine balances the activity of mitochondrial complexes I, II, and III in the TCA cycle, both in the livers of burn septic mice and in cultured hepatocytes challenged with LPS. This equilibrium in mitochondrial complex activity contributes to the amelioration of liver injury caused by burn sepsis. It is noteworthy that on the third day after the burn, the decrease in the activity of complex II was not significant, but became markedly reduced by the fifth day, showing a delayed injury to this complex. In this case, Gln seems to be less required to exert protection as the injury itself is not significant. Moreover, analysis of electron leakage from the mitochondrial respiratory chain complexes revealed that complexes I and III are considered the main sites of electron leakage [61, 62], therefore we speculate that this delayed reduction may be due to the lower electron leakage of complex II compared to complex III. While previous research has already demonstrated the hepatoprotective effects of glutamine, our findings are significant as they reveal a novel role for glutamine in directly modulating energy metabolism [29, 30].

It is widely recognized that intact and functioning electron transport chain (ETC) complexes are vital for mitochondrial electron transfer and subsequent ATP generation. Conversely, dysfunction within the complex can lead to electron leakage, resulting in the production of reactive oxygen species (ROS). These ROS, in turn, contribute to cellular and tissue injury, including energy insufficiency and lipid peroxidation [63]. Our study indicates that glutamine can reduce total ROS production and inhibit LPS-induced cell apoptosis, suggesting that glutamine promotes a stable state of mitochondrial energy metabolism, enhances ATP generation, and mitigates oxidative stress. In conclusion, our results shed light on a previously unknown aspect of glutamine's functionality, namely its direct modulation of key components within the TCA cycle. This discovery expands our understanding of glutamine beyond its role as a substrate for anaplerosis or a regulator of undesirable cellular responses.

Heat shock proteins (HSPs), also referred to as stress proteins, are remarkably conserved and play a crucial role in maintaining cellular equilibrium when confronted with various stressors, such as elevated temperatures and oxygen deprivation [64]. Among these proteins, the 60 kDa heat shock protein (HSP60) is predominantly localized within mitochondria, where it acts in concert with the 10 kDa heat shock protein (HSP10) as a co-chaperone. Together, they work to alleviate mitochondrial dysfunction and safeguard cells against the detrimental effects of ischemia/reperfusion injury [27, 28]. At a molecular level, the HSP10-HSP60 complex serves a vital function by facilitating the correct folding of misfolded proteins within the mitochondria, including those found in mitochondrial complexes II and III [16–19, 65–67]. This complex also plays a crucial role in preventing protein aggregation in various diseases, enhancing the cellular antioxidant capacity, and promoting energy metabolism. However, there is currently a lack of research on the specific relevance of the HSP10-HSP60 complex to burn sepsis. Therefore, we conducted an analysis to examine the impact of glutamine (Gln) on the folding of mitochondrial proteins following treatment with LPS. Our findings revealed that glutamine has the ability to decrease the misfolding of mitochondrial proteins and sustain the functionality of mitochondrial complexes, consequently promoting ATP production. However, when HSP60, a key component of the HSP10-HSP60 complex, was inhibited, this beneficial effect of glutamine was no longer observed. The results showed that glutamine could reduce the misfolding of mitochondrial proteins and maintain the activity of mitochondrial complexes to promote ATP production, while inhibition of HSP60 eliminated this ability. This suggests a new pharmacological property of Gln by connecting the promotion of cellular energy supply with the regulation of HSP60 assembly.

HSP60 is involved in various cellular processes, including inflammation, mitochondrial dysfunction, carcinogenesis, and cell replication. However, the precise mechanisms by which it carries out these functions as a single-gene molecule require further investigation. Some post-translational modifications (PTMs) that affect HSP60 function, such as mitochondrial dysfunction, tumor invasion, and cell apoptosis, have been studied [68–70]. In the liver, mitochondrial proteins undergo nutrient-dependent lysine acetylation and deacetylation [71, 72]. Recent research has demonstrated that SIRT3, a member of the sirtuin family, regulates the assembly of the mtHSP10-mtHSP60 chaperone protein complex by modulating the deacetylation of HSP10. This regulation plays a functional role in controlling mitochondrial complex activity [18]. In light of these findings, we conducted experiments to evaluate the acetylation levels of total mitochondrial proteins following LPS treatment. The results demonstrated that LPS increased the overall acetylation of mitochondrial proteins in AML-12 cells. However, both glutamine and pan-Acetyltransferase inhibitors (Pan-ATCi) were able to reduce the acetylation levels. Acetylation modifications play a critical role in regulating complex assembly and energy metabolism, and alterations in the acetylation levels of specific amino acids in HSP60 may contribute to the development of various diseases [65]. Consequently, we investigated the effect of glutamine on the acetylation level of HSP60. Our experimental findings revealed that LPS treatment led to an increase in the acetylation modification of HSP60, while glutamine was able to reverse this effect. These results suggest that glutamine can alleviate protein misfolding and enhance ATP production by inhibiting the acetylation of mitochondrial HSP60.

The SIRT family, which consists of NAD ^+ ^-dependent deacetylases, plays a crucial role in regulating protein deacetylation, thereby impacting aging and metabolism. Among the SIRT family members, SIRT4 stands out as a mitochondrial-specific protein with a strong affinity for NAD ^+ ^. It plays a critical role in mitochondrial energy metabolism [73, 74]. SIRT4 has been shown to promote mitochondrial function, as its deficiency reduces ATP levels while its overexpression increases ATP levels [73, 75]. It interacts with various components of the Krebs cycle and the electron transport chain, such as 2-oxoglutarate dehydrogenase (OGDH), insulin-degrading enzyme (IDE), and anion transporters, to regulate cellular energy metabolism further [73, 75]. The deacetylation activity of SIRT4 has been linked to the regulation of mitochondrial acetyl-CoA decarboxylase and acetyl-CoA synthetase, highlighting its critical role in energy metabolism [74]. However, the precise mechanisms underlying these interactions remain unclear. Notably, in proteomic studies, HSP60 has been identified as a potential target protein of SIRT4 [46–48, 76]. Yet, the regulatory patterns between glutamine, SIRT4, and HSP60, as well as their involvement in driving energy metabolism, have not been fully established. To address this, we generated SIRT4-knockdown liver cells to investigate whether SIRT4 contributes to the reduction of mitochondrial protein misfolding mediated by glutamine. The experimental results revealed that knockdown of SIRT4 reversed the reduction of mitochondrial protein misfolding induced by glutamine and abolished the ability of glutamine to enhance the activity of mitochondrial complexes II and III. This suggests that glutamine may regulate the assembly of the HSP60-HSP10 complex through its impact on SIRT4 activity. Literature analysis indicates that SIRT4 possesses activities of ADP-ribosyltransferase, thiolase, and deacetylase [74, 77]. Its deacetylase activity can modulate insulin sensitivity, regulate the activities of malonyl-CoA decarboxylase (MCD) and glutamate dehydrogenase, etc. Currently, there have been no reports on SIRT4 regulating the activity of mitochondrial complex I through deacetylation in hepatocytes, nor have there been reports on the interaction between complex I and its associated components with the HSP60-HSP10 complex, which is consistent with our findings. Additionally, there are two mechanisms by which the existing HSP60-HSP10 complex regulates mitochondrial complex activity. One is that the HSP60-HSP10 complex affects complex II activity through the enzymatic activity of medium-chain acyl-CoA dehydrogenase (MCAD) [19]. The other is that the HSP60-HSP10 complex affects complex III activity by inhibiting the degradation of UQCRC1 protein [52], thereby impacting cellular energy metabolism. Therefore, we believe that SIRT4, by influencing the acetylation modification of HSP60, regulates the assembly of the HSP60-HSP10 complex, thereby affecting the activities of complexes II and III. Given that the HSP60-HSP10 complex does not affect the activity of complex I, it is reasonable as the knockdown of SIRT4 has no effect on the activity of complex I. Additionally, knockdown of SIRT4 eliminated glutamine's ability to deacetylate lysine residues on mitochondrial HSP60 protein. These findings suggest that HSP60 may serve as a target of SIRT4. Interaction analysis further confirmed the interaction between HSP60 and SIRT4. Collectively, these findings suggest that glutamine may exert its effects on restoring electron transport chain (ETC) activity and maintaining hepatic energy metabolism by activating SIRT4 and reducing excessive acetylation modifications. This provides a novel mechanism for the role of glutamine in cellular energy homeostasis.

Studies have shown that glutamine can promote the formation of NAD + through the TCA cycle and NAD synthase [34]. Our own findings corroborate these results, showcasing how Gln can effectively boost NAD^+^ production and energy metabolism in both animal and cellular models. NAD^+^ plays a crucial role in maintaining metabolic reprograming by activating sirtuin-mediated deacetylation, regulating the activity of mitochondrial enzymes, and ensuring the proper functioning of the mitochondrial unfolded protein response. This intricate network connects energy metabolism to cellular adaptation and overall organismal response [34]. It is noteworthy that NAD^+^ acts as a coenzyme for SIRT4, influencing its deacetylase activity. Therefore, it is plausible to suggest that glutamine exerts its regulatory effects on SIRT4 through the modulation of NAD ^+ ^. By positively modulating NAD^+^ levels, we observed that glutamine promotes the activation of SIRT4, subsequently bolstering the activity of mitochondrial complexes and ATP generation. Furthermore, in vitro experiments have demonstrated that SIRT4 facilitates the assembly of the HSP60-HSP10 complex, with this effect being more prominent in the presence of NAD ^+ ^. These findings serve to reinforce the notion that the elevation of NAD^+^ levels through glutamine-induced activation of SIRT4 and promotion of HSP60-HSP10 complex assembly represents a pivotal mechanism and potential therapeutic target for maintaining optimal energy metabolism. Moreover, this knowledge holds significant implications for gaining a deeper understanding of the protective role of glutamine in burn sepsis.

## 5. Conclusions

**Figure 7.**
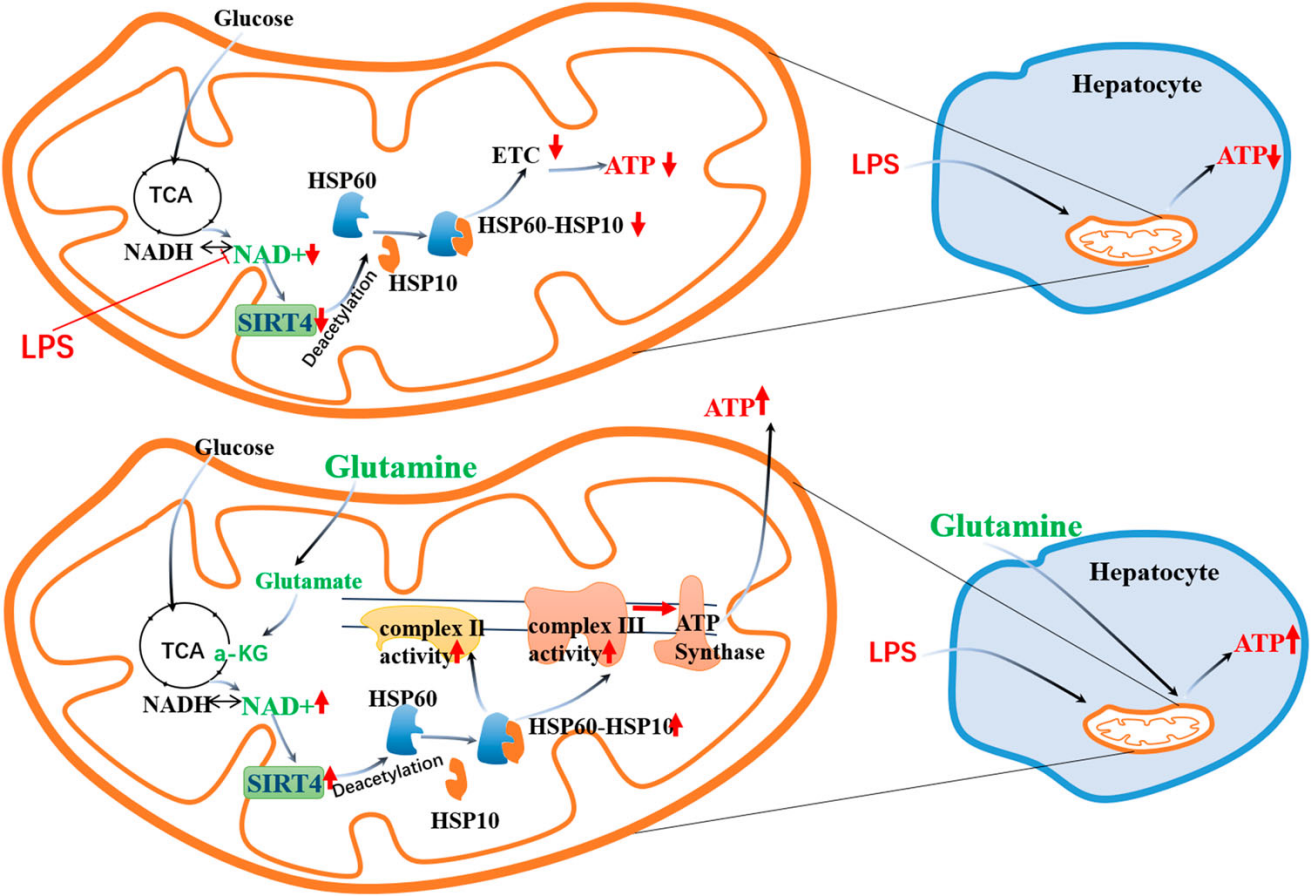
Gln regulates the acetylation modification of HSP60 through the NAD+/SIRT4 pathway, promoting the assembly of the HSP60-HSP10 complex to maintain the activity of mitochondrial complex, thereby regulating cellular energy metabolism.

We have demonstrated that Gln promotes the functional role of SIRT4 in regulating HSP60 deacetylation modification through NAD^+^, which in turn regulates the interaction of the HSP10-HSP10 complex, controlling mitochondrial protein folding and energy metabolism. This mechanism establishes a new nutritional sensing regulatory program that may contribute to the overall control of mitochondrial energy metabolism and the alleviation of organ injury in burn sepsis. Notably, Gln does not significantly affect normal cell metabolism but provides protection to cells affected by LPS. The detail mechanism has been shown in Figure 7.

## Abbreviations

Gln, glutamine; MDA, malondialdehyde; MPO, myeloperoxidase; SOD, superoxide dismutase; CAT, catalase, Pan-ATCi, Pan-Acetyltransferase inhibitors, ITC, Isothermal Titration Calorimetry; ALT, Alanine transaminase; AST, Aspartate transaminase; ATCC, American type culture collection; NMN, β-Nicotinamide mononucleotide; CO-IP, Co-immunoprecipitation; NAD^+^/NADH, Nicotinamide adenine dinucleotide; ROS, reactive oxygen species; HSP60, Heat Shock 60 kDa; HSP10, Heat Shock 10 kDa; SIRT4: Sirtuin 4; LPS: Lipopolysaccharide; COX4, Cytochrome c oxidase IV.

## Author contributions

XL and XP were responsible for trial design and process supervision. SJF, LX, and QC participated in animal experiments, specimen collection, and data acquisition. YLL, QC, QYH, and YYJ were responsible for obtaining test data. YJY and QC contributed to statistical analysis and manuscript drafting, with YJY being the primary drafter of the manuscript. XL and XP revised the manuscript. All authors participated in result discussions and provided feedback on the manuscript.

## Supplementary Material

Fig S1.tif

Fig S2.tif

## Data Availability

Data will be made available on request.
