# Supplementary figures and images for: Glutamine sustains energy metabolism and alleviates liver injury in burn sepsis by promoting the assembly of mitochondrial HSP60-HSP10 complex via SIRT4 dependent protein deacetylation
Source: Redox Rep. 2024 Feb 8;29(1):2312320. doi: 10.1080/13510002.2024.2312320 (PMC10854458; doi:10.1080/13510002.2024.2312320)

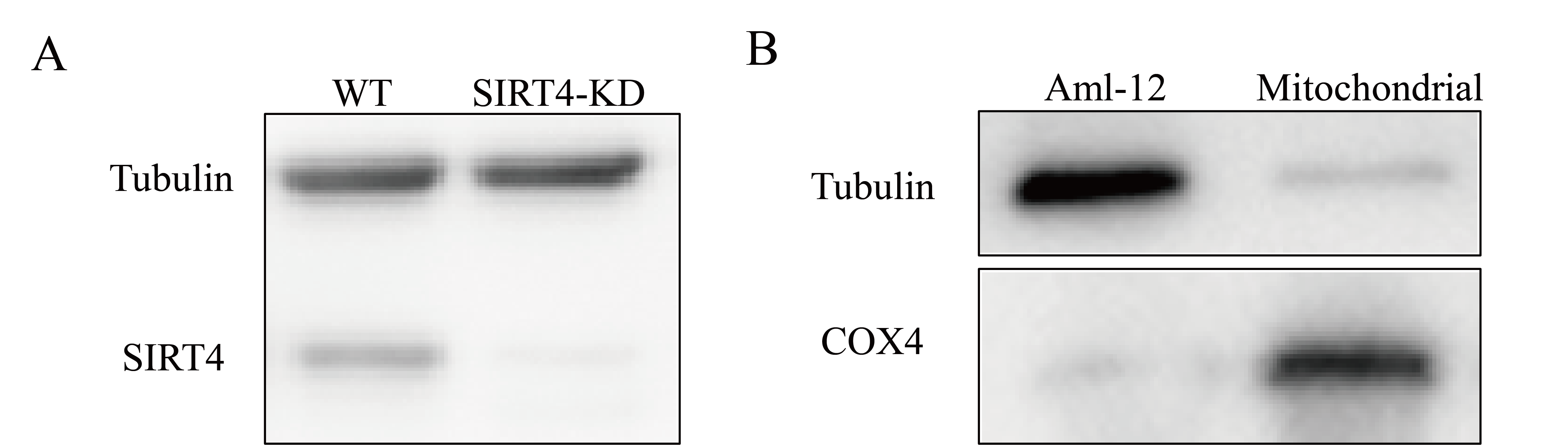

Supplement: Fig S1.tif [file YRER_A_2312320_SM0181.tif]

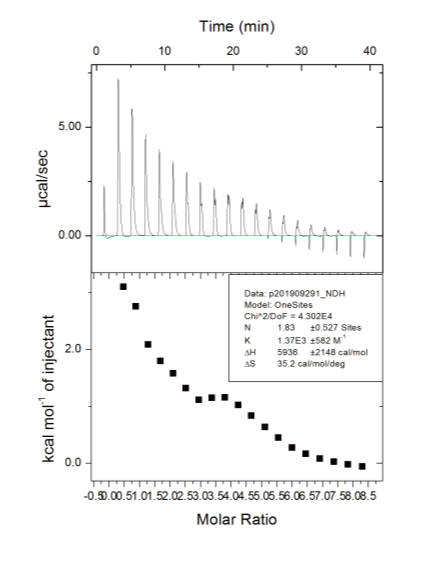

Supplement: Fig S2.tif [file YRER_A_2312320_SM0180.tif]
